# Mesenchymal Stem Cell Alterations in Bone Marrow Lesions in Patients With Hip Osteoarthritis

**DOI:** 10.1002/art.39622

**Published:** 2016-06-24

**Authors:** T. Mark Campbell, Sarah M. Churchman, Alejandro Gomez, Dennis McGonagle, Philip G. Conaghan, Frederique Ponchel, Elena Jones

**Affiliations:** ^1^ University of Ottawa, Ottawa, Ontario, Canada; ^2^ University of Leeds and NIHR Leeds Musculoskeletal Biomedical Research UnitLeedsUK

## Abstract

**Objective:**

In patients with osteoarthritis (OA), bone marrow lesions (BMLs) are intimately linked to disease progression. We hypothesized that aberrant multipotential stromal cell (also known as mesenchymal stem cell [MSC]) responses within bone tissue contributes to BML pathophysiology. The aim of this study was to investigate BML and non‐BML native subchondral bone MSCs for numeric, topographic, in vitro functional, and gene expression differences.

**Methods:**

Ex vivo 3T magnetic resonance imaging (MRI) of the femoral heads of 20 patients with hip OA was performed. MRI‐determined BML and non‐BML regions were excised and enzymatically treated to extract cells and quantify MSCs using flow cytometry and colony‐forming unit–fibroblast (CFU‐F) assay. Immunohistochemical analysis was performed to determine in vivo CD271+ MSC distribution. Culture‐expanded CD271+ cells were analyzed for tripotentiality and gene expression.

**Results:**

BML regions were associated with greater trabecular bone area and cartilage damage compared with non‐BML regions. The proportion of CD45−CD271+ MSCs was higher in BML regions compared with non‐BML regions (median difference 5.6‐fold; *P <* 0.001); the CFU‐F assay showed a similar trend (median difference 4.3‐fold; *P* = 0.013). Immunohistochemistry revealed CD271+ cell accumulation in bone adjacent to cartilage defects and areas of osteochondral angiogenesis. BML MSCs had lower proliferation and mineralization capacities in vitro and altered expression of *TNFSF11*/RANKL and *CXCR4*/stromal cell–derived factor 1 receptor. OA MSCs showed up‐regulated transcripts for *CXCR1* and *CCR6* compared with MSCs derived from healthy or osteoporotic bone.

**Conclusion:**

This study is the first to show numeric and topographic alterations in native MSCs in the diseased bone of patients with hip OA. Given the associated functional perturbation of MSCs, these data suggest that subchondral bone MSC manipulation may be an OA treatment target.

Osteoarthritis (OA) is the most common form of arthritis and a major cause of chronic pain and disability 1. As the population ages, the projected number of older adults with OA is expected to increase substantially in the next 2 decades 1. The pathophysiology of OA is complex, symptomatic treatment is often ineffective, and no licensed structure‐modifying OA drugs are currently available. Established OA involves pathology in multiple tissues, but subchondral bone plays an important role in pathogenesis and symptomatology 2, 3.

With the use of magnetic resonance imaging (MRI), subchondral bone pathology, including bone marrow lesions (BMLs) 3, 4, 5, 6, can be visualized on fluid‐sensitive MRI sequences. Such BMLs are associated with overlying cartilage pathology, pain, and progression of structural abnormalities over time 5, 7. Histologically, BMLs represent mesenchymal tissue abnormalities including bone marrow fibrosis, necrosis, swollen/dying adipocytes, and alterations in trabecular bone structure 7.

Multipotential stromal cells (also known as mesenchymal stem cells [MSCs]) are nonhematopoietic, clonogenic, multipotential cells that are present in numerous tissues 8. They have attracted great interest because of their regenerative and immunoregulatory properties, as well as their increased use in cell‐based therapies 9. However, MSC behavior in vivo and any potential contribution to the development of OA remain poorly understood 10.

MSCs are abundant in trabecular bone, where they have been observed in both perivascular and bone‐lining locations 11, 12, 13. Bone‐resident MSCs are important for bone repair and remodeling by virtue of being precursors of osteoblasts, which not only form new bone but also control osteoclast activation by producing RANKL and osteoprotegerin 14. Within BMLs, perturbations in bone MSC function may lead to abnormal bone remodeling, which could affect the overlying cartilage 3. We therefore hypothesized that subchondral bone MSCs contribute to BML pathophysiology and compared the numbers, topography, in vitro differentiation capacities, and gene expression profiles of MSCs extracted from paired BML and non‐BML regions from the same OA‐affected femoral head.

## PATIENTS AND METHODS

### Patients and cells

Patients with primary hip OA who were scheduled to undergo total hip arthroplasty (THA) were recruited from the orthopedic unit at Chapel Allerton Hospital, Leeds. All patients met the American College of Rheumatology criteria for the classification of hip OA 15. Exclusion criteria included a history of inflammatory arthritis, previous hip surgery, metastatic cancer, or disorders affecting bone. All patients gave written informed consent, and the study was approved by the National Research Ethics Committee Yorkshire and Humberside. As a control, trabecular bone was harvested from the iliac crest in 9 age‐matched patients with pelvic fracture who were otherwise healthy (median age 57 years, range 39–84 years). Femoral heads were also collected from 5 patients with osteoporosis (OP) who had a femoral neck fracture (median age 83 years, range 74–92 years).

**Figure 1:**
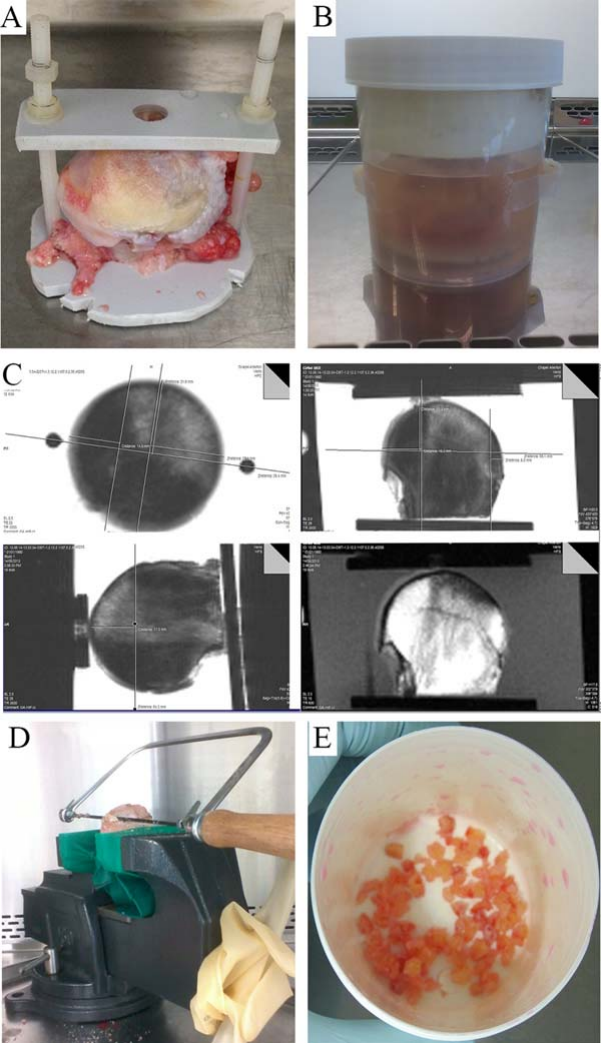
Segregation of bone marrow lesion (BML) and non‐BML regions and downstream processing. **A,** Femoral head in plastic bracket. **B,** Femoral head/bracket in a phosphate buffered saline–containing polypropylene jar. **C,** Proton density–weighted sequence magnetic resonance images in 3 planes, with measurements for cuts (top and bottom left) and corresponding T1‐weighted sequence image obtained in the “coronal” plane (bottom right). **D,** Bone segregation apparatus. **E,** BML bone fragments used for collagenase treatment after mincing with a rongeur.

Femoral heads from patients with OA or OP were collected immediately after removal during THA and placed in phosphate buffered saline (PBS) (Invitrogen). Twenty‐one femoral heads from patients with OA (median age 65.3 years, range 48–83 years) were subjected to MRI, and femoral heads from 7 patients (median age 71 years, range 43–78 years) were used as controls in real‐time quantitative polymerase chain reaction (qPCR) and flow cytometry validation experiments. For MRI, explants were mounted on a nonmetal bracket and clamp with orienting cut‐outs (Figure 1A) and secured in a PBS‐containing polyethylene jar (Figure 1B). Cod liver oil pills were fixed externally to provide further position markers. These orienting markers could be visualized on MRI and during manual processing. Samples were kept at room temperature, and MRI was performed within 4 hours of sample collection.

### MR image acquisition and analysis

MR images were obtained with a Verio 3.0T MRI system (Siemens). MRI was performed using fat‐suppressed, fast spin‐echo, proton density–weighted sequences. T1‐weighted sequences were obtained in 1 plane to identify BMLs (Figure 1C). To ensure accurate cutting measurements in 3 dimensions for separation of BML from non‐BML regions, MRI sequences were obtained in 3 planes (axial, coronal, and sagittal) (Figure 1C). BMLs were defined as areas of increased signal intensity adjacent to subcortical bone on proton density–weighted sequences that had low intensity on T1‐weighted sequences (Figure 1C). For additional information, including MRI sequence settings, see Supplementary Table 1 (available on the *Arthritis & Rheumatology* web site at http://onlinelibrary.wiley.com/doi/10.1002/art.39622/abstract).

MR images were examined using Siemens syngo fastView software for DICOM images. Identification of BML and non‐BML regions was first performed by a physiatrist with experience in musculoskeletal imaging (TMC). Interpretation was repeated independently by a radiologist with expertise in musculoskeletal imaging (RH), with blinding with regard to all patient data and prior interpretation. Consensus regarding BML and non‐BML regions was then reached by discussion. Because the patients recruited into the study had severe OA, we acknowledge that non‐BML regions are unlikely to represent “normal bone”; however, the most normal‐appearing bone was selected.

### Excision of specimens from BML and non‐BML regions, and downstream tissue processing

MRI measurements were performed in all 3 proton density–weighted sequence planes (Figure 1C). Bone cutting measurements were planned using the measuring tool of the image visualization software. Measurements and sample orientation were accomplished with reference to sample anatomic landmarks (e.g., outer cortex apex, ligamentum teres, osteophytes). A sterile marker was used to mark orienting lines on the sample corresponding to the anteroposterior and mediolateral landmarks on the bracket, so that orientation was maintained.

All bone processing was performed in a tissue culture hood, using sterile technique. After the femoral heads were removed from the bracket, they were placed in a vice, and the remainder of the femoral neck was used to hold the sample to prevent damage to the subchondral regions (Figure 1D). BML and non‐BML portions of bone were removed from the femoral head, referencing the 3‐dimensional cut plan (Figure 1D). Because BMLs have an ill‐defined border, a 2–3‐mm gap was left between BML and non‐BML regions to ensure that BML bone was well separated from non‐BML bone.

Excised pieces of BML and non‐BML regions were each divided into 2 portions: one for histology and one for cell extraction following enzymatic release 11. Samples prepared for collagenase treatment were minced using a rongeur (Figure 1E) and then placed in low‐glucose Dulbecco's modified Eagle's medium (DMEM; Life Technologies) with 20% fetal calf serum (FCS) (Sigma) and animal origin–free collagenase (3,000 units/gm bone) (Worthington Biochemical Corporation) for 4 hours at 37°C 11. After completion of the collagenase treatment, a fraction of the cells (average 1.5 × 10^5^) was obtained for flow cytometry, and the rest of the cells were frozen in FCS supplemented with 10% dimethylsulfoxide (Sigma) for later use. Samples for histologic analysis were placed in 10% formalin (Sigma) before processing.

### Histologic and immunohistochemical analysis

For each patient, separate BML and non‐BML femoral head pieces were decalcified using 12.5% EDTA (Sigma) in deionized water for 3–4 months and then mounted on paraffin blocks. Decalcified tissue specimens were stained with hematoxylin and eosin or Safranin O, using standard protocols.

**Figure 2:**
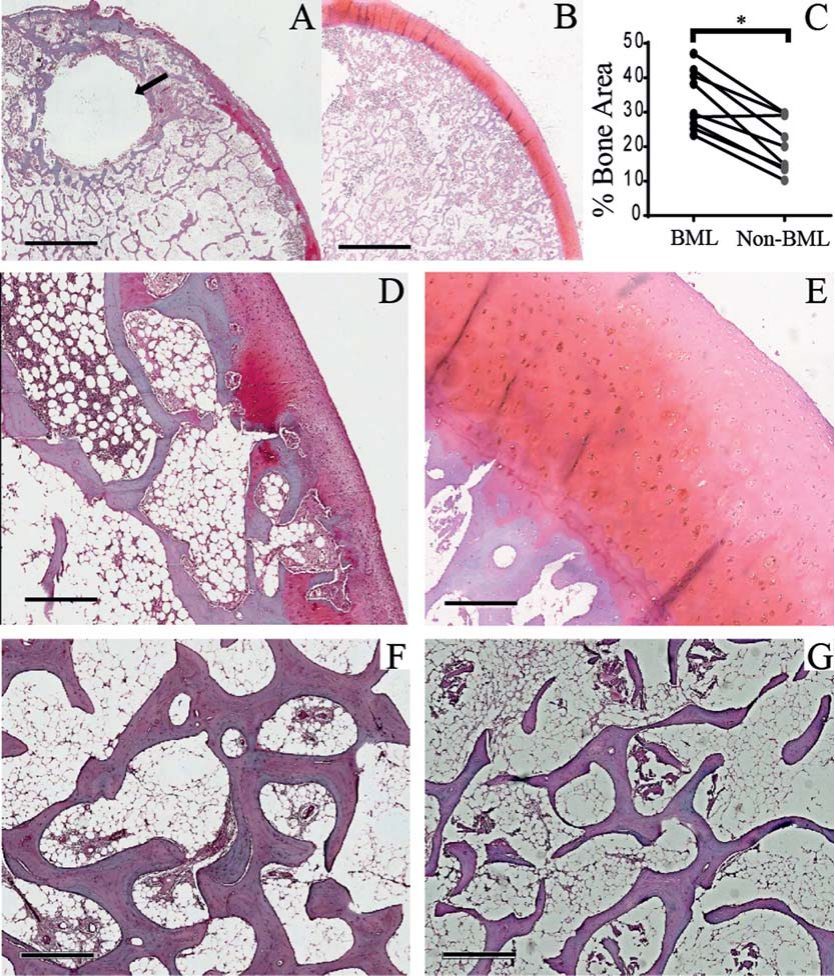
Histologic appearance of bone and cartilage in bone marrow lesion (BML) and non‐BML specimens. **A** and **B,** Gross histologic appearance of an excised BML specimen with a subchondral cyst (**arrow**) (**A**) and corresponding non‐BML specimen from the same femoral head (**B**). **C,** Comparison of trabecular bone area (BML versus non‐BML) as a percentage of the total area (n = 14 pairs). ∗ = *P* = 0.001. **D** and **E,** Excised BML specimen showing some cartilage abnormalities above the articular end plate (**D**) and non‐BML fragment from the same femoral head showing intact cartilage above the articular end plate (**E**). **F** and **G,** Photomicrographs of Safranin O–stained paired BML (**F**) and non‐BML (**G**) specimens after decalcification in EDTA, showing a greater trabecular area in the BML region. Bars = 3.8 mm (**A** and **B**), 500 μ*M* (**D** and **E**), and 600 μ*M* (**F** and **G**).

Digital image analysis was performed to evaluate relative trabecular bone area and cartilage damage. For each sample, the whole tissue area was scanned using a Nikon E1000 microscope under brightfield mode and a multispectral Nuance camera (PerkinElmer). Using the femoral head cartilage surface to orient the tissue, the overall section was then separated into cartilage (including the cartilage–bone interface) and bone. The presence of cysts in the bone area (Figure 2A) was taken into consideration, and tissue that was immediately adjacent was not used for quantitative bone area measurements. Depending on the size of the section, at least 5 (nonoverlapping) images were captured for the bone area and 2–5 images were captured for the cartilage area.

Nuance version 3.0.1.2 software (Caliper Life Sciences) was used for digital image analysis. Educating the software to recognize trabecular bone was done by manually determining small representative areas of bone and repeating the process until >95% accuracy in identifying bone was achieved automatically. For each tissue section, the full area was measured, and the trabecular bone area was calculated as a percentage (mean ± SD of a minimum of 5 images). For cartilage assessment, 10 positions were spaced out over the length of available cartilage in each image and repeated over 2–5 images (depending on the size of the tissue). Cartilage appearance was classified as “less damaged” or “more damaged” based on superficial zone smoothness, clefts, fibrillation, and presence of sclerotic bone or reparative tissue within denuded surface 16 (Figures 2D and E).

Immunohistochemistry for CD271 staining was performed as optimized by Tormin et al 12. Mouse anti‐human CD271 monoclonal antibody (Abcam) was used at a dilution of 1:50. (For a complete list of reagents, see Supplementary Table 2, available on the *Arthritis & Rheumatology* web site at http://onlinelibrary.wiley.com/doi/10.1002/art.39622/abstract).

### Flow cytometry

**Figure 3:**
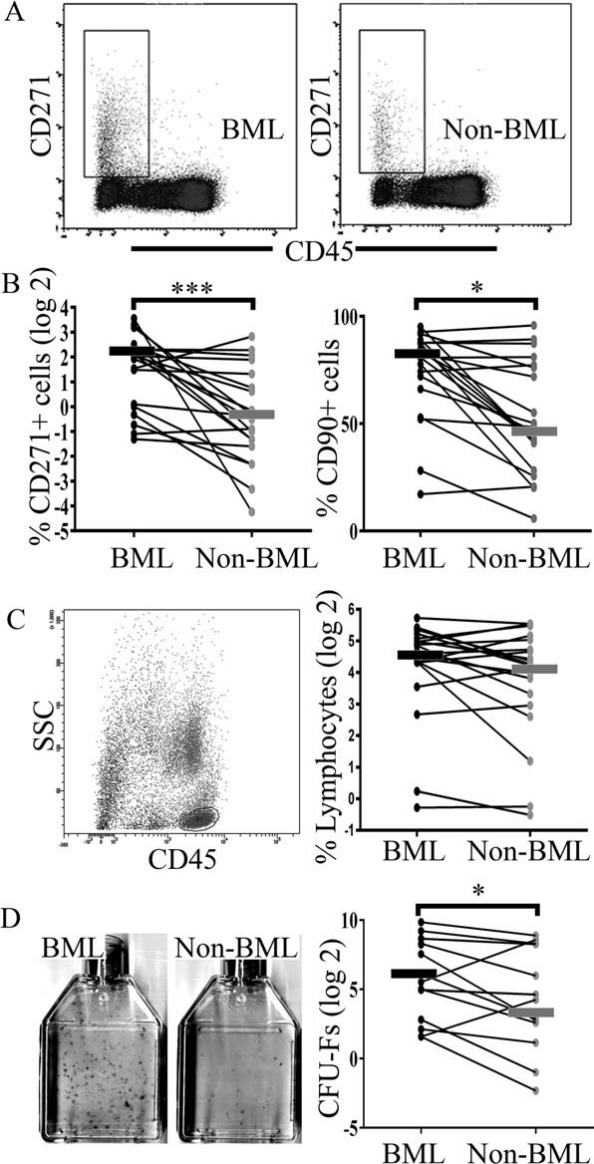
Mesenchymal stromal cell (MSC) enumeration in fractions of cells released from bone marrow lesion (BML) and non‐BML regions, following collagenase treatment. **A,** Representative flow cytometry plots for CD45−CD271+ MSC populations showing a rectangular selection for MSC enumeration. **B,** Paired‐sample line graphs showing CD45−CD271+ MSCs as a percentage of total live cells (left) and CD90+ cells as a percentage of CD45−CD271+ cells (right) in BML versus non‐BML cell fractions (n = 20 each). **C,** Left, Dot plots from a BML sample with gating on the CD45+ lymphocyte population. Right, Paired‐sample line graphs showing lymphocytes as a percentage of total live cells in BML versus non‐BML cell fractions (n = 20 each). **D,** Colony‐forming unit–fibroblast (CFU‐F) assay. Left, Representative 25‐cm^2^ flasks. Right, Paired‐sample line graph showing the number of CFU‐Fs per 10^6^ plated cells in BML and non‐BML regions (n = 14 each). In all line graphs, bars show the median. ∗ = *P* < 0.05; ∗∗∗ = *P* < 0.001.

For MSC enumeration, flow cytometry was performed on freshly enzymatically treated samples using a BD LSRII flow cytometer (BD Biosciences). Depending on the cellularity of collagenase‐treated samples, ∼1–2 × 10^5^ cells were resuspended in 50 μl of fluorescence‐activated cell sorting (FACS) buffer (PBS plus 0.5% bovine serum albumin) and incubated in a 10% Fc receptor–blocking reagent solution (Miltenyi Biotec) before fluorophore‐conjugated antibodies were added. Staining was performed for CD90, CD73, CD45, and CD271 (for additional information, see Supplementary Table 3, available on the *Arthritis & Rheumatology* web site at http://onlinelibrary.wiley.com/doi/10.1002/art.39622/abstract), and dead cells were excluded using live cell marker calcein violet and dead cell marker aqua‐fluorescent reactive dye (Invitrogen) 17. The proportion of MSCs gated as CD45−CD271+ cells 11, 12 was calculated relative to total live cells (Figure 3A). MSC extended phenotype was investigated using CD73 and CD90 markers. The percentages of lymphocytes 18 were similarly calculated relative to the total number of live cells.

The MSC identity of BML‐ and non‐BML–derived adherent cultures was investigated following staining with a standard panel of antibodies defining the phenotype of cultured MSCs 19. Passage 3 cultures grown from BML‐ and non‐BML CD271–selected cells were trypsinized and resuspended at 10^7^ cells/ml in FACS buffer. Antibody combinations included the following: phycoerythrin–Cy7 (PE–Cy7)–conjugated CD45, PerCP‐conjugated CD34, allophycocyanin (APC)–conjugated CD271, APC‐H7–conjugated CD14, and fluorescein isothiocyanate (FITC)–conjugated CD19 and PE‐labeled antibodies, including PE‐conjugated CD73 (ecto‐5′‐nucleotidase), PE‐conjugated CD105 (endoglin), and PE‐conjugated CD90 (Thy‐1) (for additional information, see Supplementary Table 3). All antibodies were used at the concentrations recommended by the manufacturers, with matched isotype controls. Dead/dying cells (normally <5% of total cells) were excluded from the analysis using 10 μl/ml DAPI (Sigma). All flow cytometry data were analyzed using Diva version 6.2 software (BD Biosciences).

### Colony‐forming unit–fibroblast (CFU‐F) assay and MSC expansion from BML and non‐BML collagenase digests

A CFU‐F assay was performed as described previously 11, with a minor modification using methylene blue, before scoring was performed in a blinded manner. MSC expansion was used to measure MSC proliferation rates and to produce a sufficient number of cells for trilineage differentiation assays and gene expression analysis. MSCs were expanded in StemMACS MSC Expansion Media after preenrichment using CD271 MACSelect MicroBeads (both from Miltenyi Biotec), and culture population doublings were calculated as previously described 20. MSCs from the bone of healthy controls and patients with OP were expanded similarly following their enzymatic release from bone 11.

### Trilineage differentiation

Passage 2/3 MSCs (n = 5 matched donor–derived cultures for BML and non‐BML bone tissue digests) were induced toward osteogenesis, chondrogenesis, and adipogenesis, using standard protocols 21. For osteogenesis and chondrogenesis, we used StemMACS OsteoDiff and ChondroDiff medium, respectively (Miltenyi Biotec); adipogenic cultures were grown in DMEM with 10% FCS, antibiotics, 10% horse serum (StemCell Technologies), 0.5 m*M* isobutylmethylxanthine, 60 μ*M* indomethacin, and 0.5 μ*M* hydrocortisone (all from Sigma).

Differentiation assessment was performed as previously described 21. Briefly, alkaline phosphatase activity was visualized on day 14 postinduction. Calcium deposits were stained using alizarin red on day 21, and total calcium produced by cultures was measured using a Calcium Detection Kit (Sentinel Diagnostics). Biochemical assessment of the glycosaminoglycans (GAGs) was performed on 3 of 4 chondrogenic pellets grown for 21 days. The remaining pellet was used for histologic analysis; 4‐μm sections were cut using a Leica CM1950 cryostat, fixed, and stained with toluidine blue. Adipogenic cultures were stained with oil red O on day 21 postinduction.

### Real‐time qPCR

To investigate MSC molecular profiles, paired CD271 bead–selected BML and non‐BML passage 2 cultures were analyzed for their relative expression of genes involved in MSC tripotentiality, collagen metabolism, chemotaxis, angiogenesis, and control of osteoclast activation. Other selected genes included those previously described as being associated with OA 3, 22 (see also Supplementary Table 4, available on the *Arthritis & Rheumatology* web site at http://onlinelibrary.wiley.com/doi/10.1002/art.39622/abstract). MSCs derived from trabecular bone from age‐matched controls as well as MSCs derived from the femoral heads of patients with OP were included as controls.

Reverse transcription and qPCR were performed using a custom Format 48 TaqMan low‐density array (Life Technologies) (see Supplementary Table 4), as previously described 20. Mean fold changes were calculated and were considered further if the change was ≥2‐fold. Selected transcripts were validated on additional samples using individual TaqMan assays, matching those included in the TaqMan low‐density array. Tests were performed in triplicate on 5 ng complementary DNA per well.

### Statistical analysis

Differences between paired BML and non‐BML samples for trabecular bone area, flow cytometric measurements of MSC and lymphocyte proportions, CFU‐F assays, MSC growth, gene expression, and differentiation data were compared using the 2‐sample paired sign test. Gene expression differences using control MSC cultures (3 groups: OA, healthy control, and OP MSCs) were tested by Kruskal‐Wallis test with Dunn's multiple comparison tests. The chi‐square test was used to establish associations between cartilage appearance (less damaged versus more damaged) and BML/non‐BML images. *P* values less than or equal to 0.05 were considered significant. All tests were performed using IBM SPSS Statistics 21.

## RESULTS

### Subject recruitment

Twenty‐one patients were considered for recruitment into the ex vivo MRI study, but 1 of the patients showed no detectable BMLs on MRI and was excluded. Half of the patients were women, and all were white. The median age was 65.3 years (range 48–83 years), and the median body mass index was 28.4 kg/m^2^ (range 20.7–42.8). Half of the patients were receiving nonsteroidal antiinflammatory drugs, and none were receiving bisphosphonates. Twenty‐five percent of the patients were smokers, and 85% had Kellgren/Lawrence grade 4 hip OA 23.

### Histologic features segregating BML from non‐BML regions

To confirm the accuracy of our BML sampling, we performed histologic assessment of bone and cartilage in paired BML and non‐BML samples from 14 randomly selected patients. BMLs have been characterized by a high bone volume fraction 24. For trabecular area analysis, 320 images of bone were acquired: 198 images for BML samples (average of 9.3 images per sample [range 5–14]) and 122 images for non‐BML samples (average of 6.6 images per sample [range 5–9]). The average trabecular bone area per sample (n = 14 pairs) was widely distributed between the patients; however, a clearly higher trabecular area was observed in BML compared with paired non‐BML samples (*P* = 0.001) (Figures 2C, F, and G).

BMLs were also associated with overlying cartilage defects 25, 26. Cartilage assessment was performed in the group of samples from 14 donors. Following decalcification, 9 of 14 paired tissue samples had sufficient amounts/quality of cartilage to enable paired analysis, and 74 images were acquired for the cartilage region: 43 BML (average of 5 images per sample [range 2–9]) and 31 non‐BML (average of 3 images per sample [range 2–14]). Images showing more damaged cartilage (assessed by comparing the relative smoothness of cartilage, presence of clefts, fibrillation, and sclerotic bone or reparative tissue within denuded surface [16]) were closely associated with BML samples (24 of 43 images) compared with non‐BML samples (5 of 31 images; *P* = 0.01), where most of the cartilage surface was considered to be less damaged (Figures 2D and E). Taken together, these findings were consistent with the expected histologic features of BML and non‐BML regions, confirming the accuracy of the excision method.

### MSC enumeration by flow cytometry and CFU‐F assay

Representative flow cytometry plots for CD45−CD271+ MSC populations are shown in Figure 3A. Despite broad donor‐to‐donor variation, a greater proportion of CD45−CD271+ cells as a percentage of total live cells was observed in BML compared with non‐BML tissue digests (median difference 5.6‐fold; *P* < 0.001) (Figure 3B). The expression of 2 additional markers (CD73 and CD90) was also assessed on CD45−CD271+ cells to confirm their MSC identity. In both BML and non‐BML regions, >85% of CD45−CD271+ cells were CD73+ (for BML, mean 87.5%; for non‐BML, mean 89.5% [*P* = not significant]). The CD45−CD271+CD90+ cell subpopulation, a recently described phenotype of the most clonogenic MSCs 27, was also higher in BML regions (median 1.7‐fold; *P* = 0.041) (Figure 3B). In contrast to the observed differences in MSC numbers, no difference in the percentages of lymphocytes (gated as CD45^bright^SSC^low^ cells [18]) in BML and non‐BML digests was observed (*P* = 0.824) (Figure 3C). To further validate this difference in MSC frequency, a CFU‐F assay was performed in 14 pairs of tissue digests, and the same trend was observed (median difference 4.3‐fold; *P* = 0.013) (Figure 3D).

### Topography of CD271+ MSCs in excised BML and non‐BML specimens

**Figure 4:**
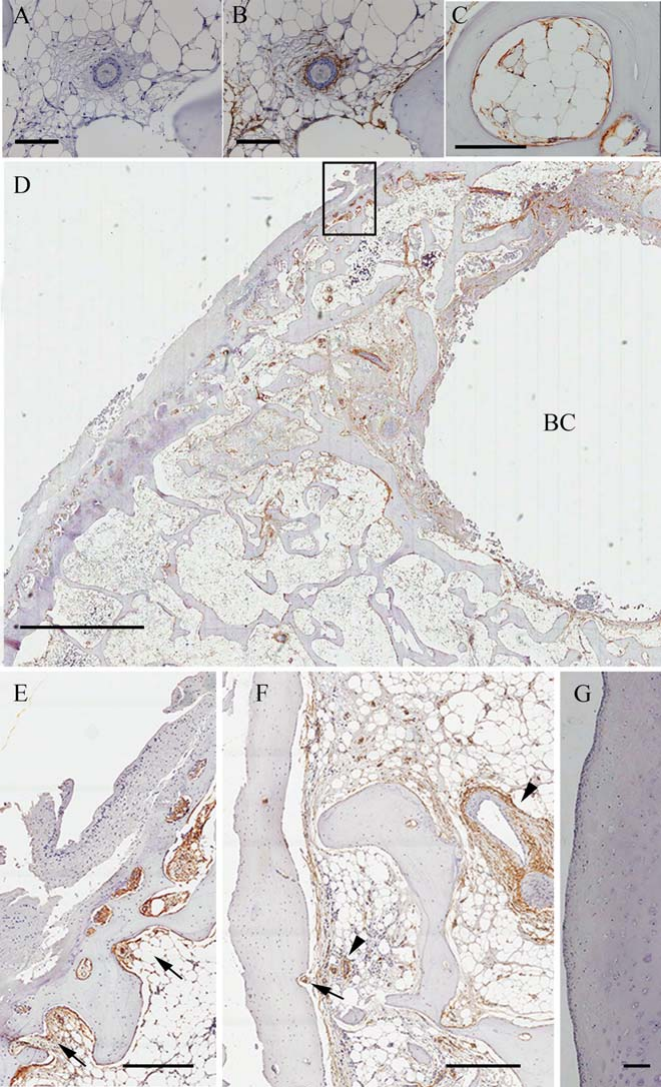
CD271 cell distribution in light microscopy images of bone marrow lesion (BML) and non‐BML specimens as assessed by immunohistochemistry. **A** and **B,** Perivascular distribution in representative negative control (**A**) and CD271‐stained (**B**) non‐BML specimens. **C,** Representative non‐BML specimen showing CD271 staining in bone lining. **D,** CD271+ staining near osteochondral junction and surrounding a bone cyst (**BC**) in a representative BML sample. **E,** Higher‐magnification view of boxed area in **D**, showing CD271+ staining within the subarticular end plate immediately beneath a chondral lesion (**arrows**). **F,** High‐magnification view of BML sample, showing subchondral CD271+ perivascular staining (**arrowheads**) and CD271+ staining within subarticular end plate immediately beneath chondral lesion (**arrow**). **G,** Non‐BML sample, showing relatively intact cartilage and lacking CD271 expression. Bars = 100 μm (**A–C**), 3 mm (**D**), 400 μm (**E** and **F**), and 200 μm (**G**).

We next performed immunohistochemical analysis to investigate the localization of CD271+ cells within the excised samples. In both BML and non‐BML tissues, CD271 staining was distributed as expected in a perivascular and reticular pattern in marrow cavities (Figure 4B) 12, 13. Additionally, CD271 positivity was clearly detectable in bone lining locations (Figure 4C).

A highly heterogeneous distribution of CD271 positivity (due to the large intersubject heterogeneity of bone pathology and cartilage OA architectural changes) did not allow reliable quantification of CD271+ cells using digital imaging analysis to directly compare excised non‐BML and BML specimens. However, in the BML samples, accumulation of CD271 staining was particularly evident in the regions adjacent to subchondral bone cysts (Figure 4D) and cartilage damage (Figures 4D–F) at osteochondral junctions where overlying cartilage loss was more pronounced. In addition to perivascular staining (Figure 4F), there was substantial staining of fibrous stromal tissue extending toward and up to the cement line from the subchondral bone (Figures E and F), suggesting that MSCs had accumulated at regions of cartilage damage. Stained stromal tissue was often seen invading more damaged cartilage “from below” in BMLs (see Supplementary Figures 1A and B, available on the *Arthritis & Rheumatology* web site at http://onlinelibrary.wiley.com/doi/10.1002/art.39622/abstract); however, there was no positive CD271+ staining within the cartilage itself (Figure 4G).

### In vitro growth and differentiation capacities of CD271+ MSC–derived cultures

**Figure 5:**
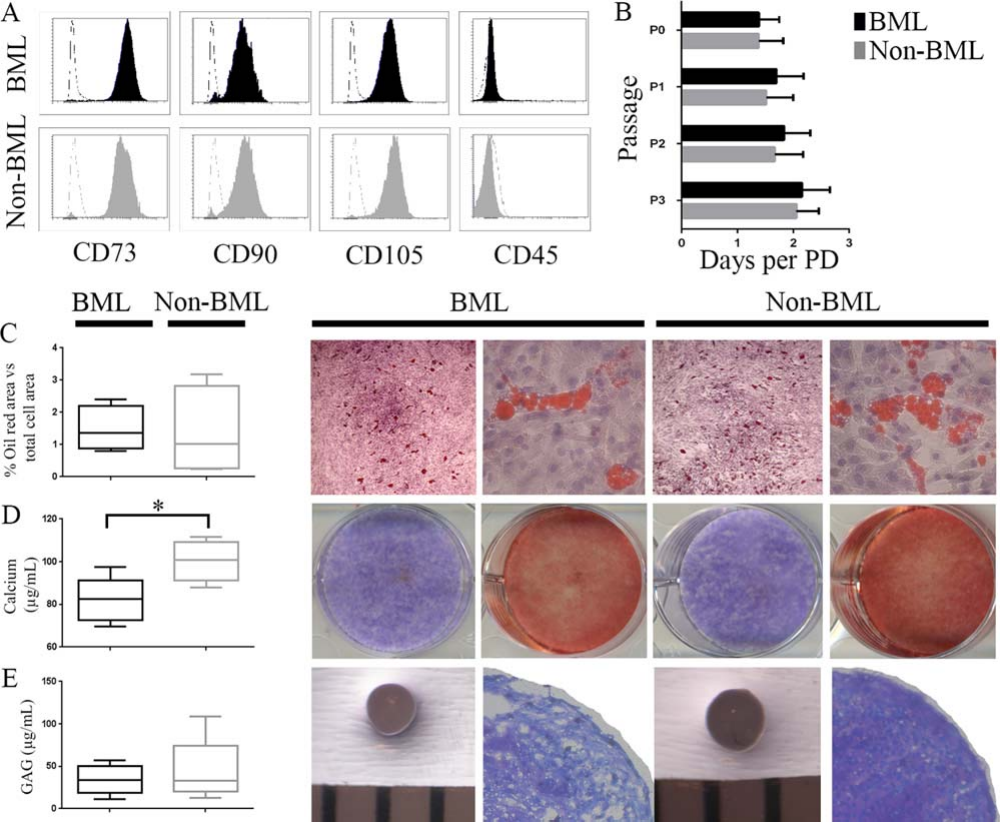
Surface phenotype, growth rates, and differentiation capacities of CD271 cell–derived cultures from bone marrow lesions (BMLs) and non‐BMLs. **A,** Flow cytometry histograms showing positive (CD73, CD90, CD105) and negative (CD45) marker expression in BMLs and non‐BMLs from a representative donor. Dashed lines indicate isotype controls. **B,** Culture growth rates at different passages (for passages 0–2 [P0–P2], n = 5; for P3, n = 2). Bars show the mean ± SD. **C,** Adipogenesis in paired BML and non‐BML adipogenic cultures. Left, Percentage of oil red O–positive area versus total cell area in BML and non‐BML cultures (n = 5 each). Right, Representative photomicrographs of paired BML and non‐BML cultures at 40× magnification and 200× magnification (left and right images, respectively). **D,** Osteogenesis in paired BML and non‐BML osteogenic cultures. Left, Calcium production in BML and non‐BML osteogenic cultures (n = 5 each). Right, Representative alkaline phosphatase (purple) and alizarin red (red) staining of BML and non‐BML osteogenic cultures. **E,** Chondrogenesis in paired BML and non‐BML chondrogenic cultures. Left, Glycosaminoglycan (GAG) production in BML and non‐BML chondrogenic cultures (n = 5 each). Right, Gross images of wet chondrogenic pellets (bars represent 1‐mm spacing) and light microscopy images of toluidine blue–stained cartilage pellets at 40× magnification. Data in **C**–**E** are shown as box plots, representing the 25th, 50th, and 75th percentiles. ∗ = *P* < 0.05. PD = population doubling.

CD271+ bead–selected cells from BML and non‐BML digests possessed the standard MSC phenotype following culture expansion (Figure 5A). To test whether BML‐resident CD271+ MSCs had altered functional capacities, the growth kinetics of CD271+ cell cultures were examined. BML cultures had slightly longer population doubling times compared with non‐BML cultures (*P* = 0.049 for all passages combined; n = 5 donors) (Figure 5B).

Regarding their differentiation capabilities, paired BML and non‐BML cultures showed similar levels of adipogenesis (Figure 5C). No differences were observed in alkaline phosphatase staining on day 14 postinduction of osteogenesis (Figure 5D); however, on day 21 postinduction, BML cultures produced lower amounts of calcium (*P* = 0.043) (Figure 5D). No obvious trends were observed in chondrogenesis assays, assessed either qualitatively (chondrogenic pellet staining with toluidine blue) or quantitatively (GAG assay) (Figure 5E).

### Comparative gene expression signatures of MSCs from BML and non‐BML digests

**Figure 6:**
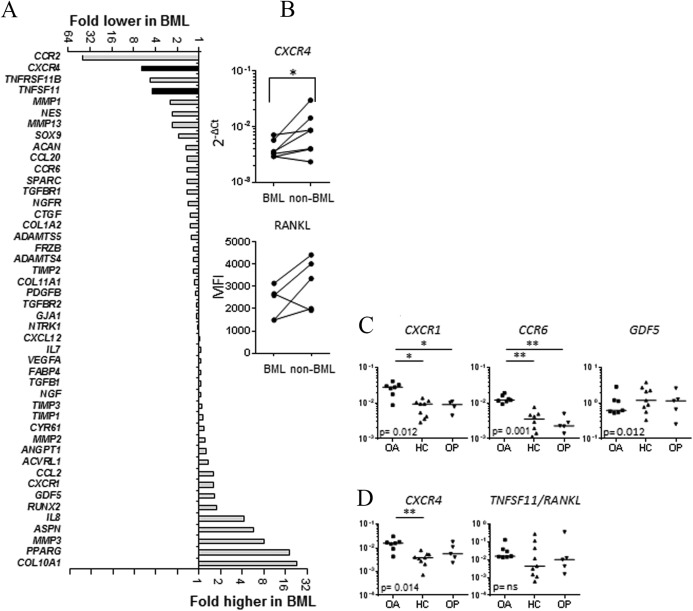
Relative gene expression in CD271+ cell–derived mesenchymal stem cell cultures from bone marrow lesions (BMLs) and non‐BMLs in the bone of patients with osteoarthritis (OA), healthy controls (HCs), and patients with osteoporosis (OP). **A,** Mean fold changes in relative gene expression in BML versus non‐BML cultures; n = 7 paired samples. The $2^{-\Delta C_{t}}$ value was normalized to the value of *HPRT*. Black bars indicate *P* < 0.05 by Wilcoxon's test for paired data. **B,** Top, Validation of *CXCR4* differential expression as determined by TaqMan quantitative polymerase chain reaction (qPCR) analysis. Bottom, Validation of RANKL surface protein expression as determined by flow cytometry. **C,** Validation of genes putatively specific for OA mesenchymal stem cells (MSCs) using additional OA (n = 7), HC (n = 9), and OP (n = 5) MSC cultures. **D,** Expression of *CXCR4* and *TNFSF11* (encoding RANKL) in additional OA, HC, and OP MSC cultures. Gene expression that is significantly different in OA MSCs compared with both HC MSCs and OP MSCs is indicative of an OA phenotype. The y‐axis indicates expression relative to *HPRT*. Kruskal‐Wallis grouped comparison *P* values are shown in graphs, with paired significance indicated by *P* < 0.05 (*) and *P* < 0.01 (**). MFI = mean fluorescence intensity; NS = not significant.

The expression of 46 genes involved in MSC function, collagen metabolism, chemotaxis, angiogenesis, and control of osteoclast activation was measured using qPCR in CD271+ cell–derived cultured MSCs from OA patients (n = 7 BML/non‐BML pairs). The 2 differentially expressed bone‐related genes between BML and non‐BML cultures (*CXCR4* and *TNFSF11*) (Figure 6A) were subsequently validated by individual TaqMan assays and flow cytometry. Consistent with TaqMan low‐density array data, expression of the receptor for stromal cell–derived factor 1 (SDF‐1), *CXCR4*, was lower in BML MSCs, using qPCR (Figure 6B). CXCR4 surface protein was present only in a small percentage 28 of cells in all 5 paired BML/non‐BML MSC cultures (see Supplementary Figure 2A, available on the *Arthritis & Rheumatology* web site at http://onlinelibrary.wiley.com/doi/10.1002/art.39622/abstract). The mean fluorescence intensity of the surface protein RANKL (encoded by *TNFSF11*) was lower in 4 of 5 cultures of BML MSCs compared with non‐BML MSCs, as demonstrated using flow cytometry (Figure 6B). The remaining genes were not differentially expressed between BML and non‐BML digests.

### Comparative gene expression signatures of MSCs from OA, OP, and healthy bone

To determine whether differential MSC gene expression in femoral heads was a feature of hip OA, we compared all OA MSC transcripts (averaging BML and non‐BML TaqMan low‐density array data for the genes that were not differentially expressed) with healthy control MSCs (n = 5) and OP MSCs from femoral heads (n = 3). When OA MSCs were compared with healthy control MSCs, significantly different levels were observed for 8 transcripts. Transcript levels for 5 of these transcripts were higher in OA MSCs: *CXCR1*/interleukin‐8 (IL‐8) receptor α‐chain and *CCR6*/macrophage inflammatory protein 1α (MIP‐1α) receptor (mostly below detection in healthy control MSCs), *GDF5*/growth differentiation factor 5 (8‐fold), *MMP1*/matrix metalloproteinase 1 (23‐fold), and *TGFBR2*/transforming growth factor β receptor 2 (2‐fold). The levels of another 3 transcripts were lower: *ACAN*/aggrecan (2‐fold), *NTRK1*/high‐affinity nerve growth factor receptor (10‐fold), and *NGFR*/low‐affinity nerve growth factor receptor (2‐fold). We next used qPCR (Figure 6C) and flow cytometry (see Supplementary Figure 2B, available on the *Arthritis & Rheumatology* web site at http://onlinelibrary.wiley.com/doi/10.1002/art.39622/abstract) to validate some of these putative OA‐specific genes, using additional cultures of OA MSCs (n = 7), and compared expression with that in healthy control MSCs (n = 9). We were mindful that these differences could be attributable to OA MSCs and healthy control MSCs having been derived from anatomically different bones (femoral head versus iliac crest 29); therefore, we analyzed OP MSCs (n = 5), which were also derived from femoral head bone. This analysis confirmed that expression of *CXCR1*, *CCR6*, and *GDF5* was significantly different across the 3 groups (Figure 6C), and that *CXCR1* and *CCR6* were OA‐specific (as shown by significantly higher expression in OA MSCs compared with both healthy control MSCs and OP MSCs). CXCR1 surface protein expression was also higher in OA MSCs compared with healthy control MSCs and OP MSCs, and shown using flow cytometry (see Supplementary Figure 2B, available on the *Arthritis & Rheumatology* web site at http://onlinelibrary.wiley.com/doi/10.1002/art.39622/abstract).

Finally, we investigated whether the expression of *CXCR4* and *TNFSF11* was OA‐specific (Figure 6D). Using additional OA, healthy control and OP MSC cultures, we observed that *CXCR4* expression was significantly different across the 3 groups and higher in OA MSCs compared with healthy control MSCs (Figure 6D). *TNFSF11*/RANKL expression was variable across the 3 groups, as demonstrated by the results of qPCR (Figure 6D) and flow cytometry, respectively (Supplementary Figure 2B).

## DISCUSSION

Relatively little is known about the role of endogenous MSCs in OA‐related bone pathophysiology 10. Given that MSCs are thought to be master regulators of joint and bone homeostasis 30, we investigated whether they might be involved in OA BMLs, which is known to be associated with both pain and structural changes 31. To our knowledge, this study is the first to evaluate native subchondral bone MSCs in human OA in relation to tissue damage. Our findings show numeric, topographic, gene expression, and functional perturbations in MSCs from patients with hip OA, especially from areas of cartilage loss in BMLs.

Previous studies investigated OA MSCs from anatomic sites remote from damaged areas, i.e., from iliac crest bone marrow 32 and from femoral canal bone marrow 33, both after culture expansion. Our previous analysis of CD45−CD271+ cells sorted from whole OA femoral heads did not reveal any significant signs of premature aging or gross osteogenic abnormality compared with control bone 11. In the current study, we carefully excised BML and non‐BML areas of a femoral head, as segregated using MRI, and were able to detect subtle differences in MSC features within the same affected joint but in relation to the amount of tissue damage. The histologic features of excised BML and non‐BML regions in our study were consistent with anticipated tissue abnormalities in BMLs, such as an increased bone volume fraction 24 and overlying cartilage loss 25, 26.

We first showed that MSCs were proportionally increased in more diseased OA bone; this was initially surprising but not entirely unexpected considering previously published reports of the increase in synovial fluid MSCs in relation to OA severity 34, 35. Consistent with these findings, Harris et al recently documented aberrant MSC accumulation in the joints of patients with advanced OA 36. Additionally, an increase in subchondral bone MSCs was recently documented in a mouse anterior cruciate ligament transection model of OA 37.

In accordance with data from the studies by Harris et al and Zhen et al and with their proposed mechanism for increased MSCs in OA joints 36, 37, we observed that the OA MSC chemokine receptor transcript profile was consistent with the notion of their potential recruitment from deeper marrow cavities toward the joint surface. BML MSCs may indeed be recruited to more damaged areas of cartilage and superficial subchondral bone due to higher concentrations of SDF‐1 in these regions, which is the result of diffusion to subchondral bone from OA synovial fluid via thinned, damaged cartilage 38, 39. Our data suggest that once at the site of damage, MSC *CXCR4* expression may be down‐regulated to prevent further migration.

Furthermore, OA MSCs up‐regulated *CXCR1* (receptor for IL‐8) and *CCR6* (receptor for MIP‐3α), 2 chemokines that are known to be abundant in OA synovial fluid 36, 40 and have been shown to be potent inducers of bone marrow MSC migration 41. Therefore, both the gene expression data and the immunohistochemical staining pattern, where MSCs were abundant in regions underlying cartilage defects, support the notion of their migratory response 42 toward areas of cartilage loss where the influences of inflammatory synovial fluid chemokine gradients are the strongest. In progressive OA, however, this response appears to be inadequate, pointing toward the possibility of a defect in MSC recruitment following skeletal damage.

In the current study, a lower MSC calcium production capacity of BML MSCs compared with non‐BML MSCs was observed, which could explain histologic findings of reduced tissue mineral density in BML bone despite a higher cross‐sectional bone area. Inappropriate mineralization of BML bone could also be attributable to the defect in the capacity of BML MSCs to regulate bone remodeling. Compared with non‐BML MSCs, BML MSCs expressed less RANKL surface protein in 4 of 5 matched MSC cultures tested. Shifts in RANKL expression, at both the messenger RNA and protein levels, have been previously documented for OA subchondral bone osteoblasts and explained by their “different stages of attempts to repair” 43. This further supports the concept of “uncoupled” bone formation and resorption by subchondral bone in OA 37, conceivably altering the biomechanical and load‐distribution properties of OA bone, putting cartilage at higher risk of injury. Such alterations support the need for further development of novel therapies targeting subchondral bone homeostasis for the treatment of OA 2, 44, 45, 46. In this context, our findings indicate that the MSC population is affected by the OA process and may therefore be an important therapeutic target for modulation in early disease.

Our histologic data showed that in the femoral heads of patients with OA, CD271+ MSCs surrounded vessels that had penetrated up to the cement line. MSCs can indeed act as promoters of angiogenesis 10 and are closely associated with pericytes and catecholaminergic nerve fibers 13, 47. OA neurovascular changes at the osteochondral junction, including vessels and both sensory and sympathetic nerves breaching the tidemark, are now considered to be a possible source of OA joint pain 48, 49. Based on our immunohistochemistry data, it is not unreasonable to suggest that MSCs in patients with advanced OA could also take part in pathologic subchondral neurovascular ingrowth (via their angiogenic actions and vessel‐stabilizing functions) and hence contribute to the development of joint pain.

This study is limited by the number of OA patients recruited for the MRI study and the amount of material that could be distributed to all of the experimental arms. Although the study was sufficiently powered to detect numeric and functional differences in paired BML/non‐BML MSC populations, some statistical analyses, such as gene expression validation using flow cytometry and TaqMan qPCR, were not possible in all cultures. Although the expression of *GDF5*, a growth factor and known OA susceptibility gene 22, 50, was found to be different in OA MSCs compared with healthy control MSCs and OP MSCs, further work is required to assess its role in influencing MSC activity at the site of damage. This is in contrast to our data for *CXCR1* and *CCR6*, the expression of which was confirmed to be OA‐specific. Finally, although we were able to observe a difference in CD271 immunohistochemical staining distribution between BML and non‐BML samples, tissue architectural heterogeneity prevented us from making a statistical evaluation of these data and comparing it with our flow cytometry findings.

In summary, our data show that in subchondral bone from patients with late‐stage hip OA, MSCs are increased in number in the areas of damage but exhibit functional and gene expression perturbations that could lead to further damage escalation. In relation to the development of novel therapy for early OA, our work emphasizes the abundance of subchondral bone MSCs in humans and provides initial insight into potential candidate pathways that can be targeted in order to normalize or improve the MSC pool. New therapies targeting the bone–cartilage interface 14 and aimed at reestablishment of a functional cartilage surface zone 10 could delay progression of the disease, particularly if they are combined with other interventions such as correction of joint biomechanics.

## AUTHOR CONTRIBUTIONS

All authors were involved in drafting the article or revising it critically for important intellectual content, and all authors approved the final version to be published. Dr. Jones had full access to all of the data in the study and takes responsibility for the integrity of the data and the accuracy of the data analysis.

### Study conception and design

Campbell, Churchman, Ponchel, Jones.

### Acquisition of data

Campbell, Churchman, Gomez, Ponchel, Jones.

### Analysis and interpretation of data

Campbell, Churchman, McGonagle, Conaghan, Ponchel, Jones.

## Supporting information


**Supplementary Table 1.** MRI Sequence Setting for Femoral Head Imaging
**Supplementary Table 2.** Reagents used for immunohistochemistry
**Supplementary Table 3.** Antibody Conjugates and Markers Used for Flow Cytometry
Supplementary Table 4 Assays used in the Taqman low density array (TLDA)

**Supplementary Figure 1** CD271 cell distribution in BML sections assessed by immunohistochemistry. Light microscopy photomicrographs. (A) BML section showing cartilage fissuring and thinning with prominent subchondral CD271^+^ staining. (B) High‐magnification image of rectangular area in (A) showing CD271^+^ staining within subarticular end‐plate immediately beneath chondral lesion. Magnification bars: 500 μm (A), 200 μm (B).

**Supplementary Figure 2** Surface expression of CXCR4, CXCR1 and RANKL proteins by flow cytometry. Cultures were grown from magnetically‐selected CD271^+^ cells. Live MSCs were gated as DAPI‐negative, CD45^‐^CD73^+^CD90^+^ cells. Histograms for representative cultures are shown for the different markers. (A) BML (empty histograms) and non‐BML (filled histograms) donor‐matched cultures and (B) MSCs cultures from HC, OP, MSC femoral heads. BML = bone marrow lesion; HC = healthy control; MSC = mesenchymal stem cell; OA = osteoarthritic; OP = osteoporotic.
