# Supplementary material for: Mesenchymal Stem Cell Alterations in Bone Marrow Lesions in Patients With Hip Osteoarthritis
Source: Arthritis Rheumatol. 2016 Jun 24;68(7):1648–59. doi: 10.1002/art.39622 (PMC4941540; doi:10.1002/art.39622)
Supplement: Supplementary file 1 — Supplementary Table 1. MRI Sequence Setting for Femoral Head Imaging Supplementary Table 2. Reagents used for immunohistochemistry Supplementary Table 3. Antibody Conjugates and Markers Used for Flow Cytometry Supplementary Table 4 Assays used in the Taqman low density array (TLDA) [file ART-68-1648-s001.docx]

**SUPPLEMENTARY MATERIALS AND METHODS**

**Supplementary Table 1.** MRI Sequence Setting for Femoral Head Imaging

|  | PDFS (3 planes) | T1W | DESS |
| --- | --- | --- | --- |
| Repetition time (msec) | 3500 | 600 | 16.3 |
| Time to echo (msec) | 29 | 15 | 4.7 |
| Slice thickness (mm) | 2.5 | 2.5 | 0.5 |
| Interslice gap (mm) | 0 | 0 | 0 |
| Field of view (mm) | 420 | 380 | 192 |
| Matrix (pixels) | 576X576 | 384X384 | 384X384 |
| Number of excitations | 1 | 1 | 1 |

PDFS = proton density–weighted sequences; T1W = T1-weighted sequences; DESS = Dual-echo steady state sequences.

**Supplementary Table 2.** Reagents used for immunohistochemistry

| Reagent/Antibody | Manufacturer |
| --- | --- |
| Reagents |  |
| EnVision Dual Endogenous Enzyme Block for peroxidases | Dako UK Ltd, Cambridgeshire, UK |
| Antibody Diluent for prevention of non-specific primary antibody binding | Dako UK Ltd |
| Harris’ haematoxylin | CTL Scientific Supply Corp, NY, USA |
| DPX mountant | Sigma-Aldrich |
|  |  |
| Primary Antibody |  |
| Mouse anti-human CD271 monoclonal primary antibody | Abcam, Cambridge, UK |
|  |  |
| Secondary antibody |  |
| Two-step anti-rabbit horseradish peroxidase micropolymer conjugate (Labelled Polymer-HRP) | Dako UK Ltd |

HRP = horse radish peroxidase

**Supplementary Table 3.** Antibody Conjugates and Markers Used for Flow Cytometry

| Target | Fluorochrome | Clone | Manufacturer |
| --- | --- | --- | --- |
| Enumeration |  |  |  |
| CD271 (Low-affinity nerve growth factor receptor) | APC | ME20.4-1.H4 | Miltenyi Biotec |
| CD45 (Leukocyte Common Antigen) | PE-Cy7 | HI30 | BD Pharmingen |
| CD73 (5′ Ecto-nucleotidase) | PE | AD2 | BD Pharmingen |
| CD90 (Thy1) | FITC | F15-42-1 | Serotec |
| Phenotyping |  |  |  |
| CD73 (5′ Ecto-nucleotidase) | PE | AD2 | BD Pharmingen |
| CD73 (5′ Ecto-nucleotidase) | BV421 | AD2 | BD Pharmingen |
| CD90 (Thy1) | PE | MCA90 | Serotec |
| CD90 (Thy1) | Alexa 700 | SE10 | Biolegend |
| CD105 (Endoglin) | PE | SN6 | Serotec |
| CD19 (B lymphocyte antigen) | FITC | HIB19 | BD Biosciences |
| CD34 (gp105–120) | PerCP | 8G12 | BD Biosciences |
| CD14 (Monocyte antigen) | APC-H7 | M5E2 | BD Biosciences |
| CD45 (Leukocyte common antigen) | PE-Cy7 | HI30 | BD Biosciences |
| Protein expression |  |  |  |
| CXCR1/CD181 | FITC | 8F1/CXCR1 | Biolegend |
| CXCR4/CD184 | PE-Cy5 | 12G5 | BD Pharmingen |
| RANKL/CD254 | PE | MIH24 | Biolegend |
|  |  |  |  |
| Isotype controls |  |  |  |
| IgG1 | FITC | MOPC-31C | BD Biosciences |
| IgG1 | PE | MCA928PE | Serotec |
| IgG1 | PerCP | MOPC-21 | BD Biosciences |
| IgG1 | PE-Cy7 | MOPC-21 | BD Biosciences |
| IgG2a | APC-H7 | G155-178 | BD Biosciences |
| Live/Dead Markers |  |  |  |
| Live Marker | Calcein violet acetoxymethyl | N/A | Invitrogen |
| Dead Marker | Aqua-fluorescent reactive dye | N/A | Invitrogen |
| DAPI | DAPI | N/A | Sigma |

APC = Allophycocyanin; Cy7 = Cyanine 7; DAPI = 4',6-diamidino-2-phenylindole; FITC = Fluorescein isothiocyanate; PE = R-phycoerythrin; PerCP = Peridinin Chlorophyll.

Supplementary Table 4 Assays used in the Taqman low density array (TLDA)

| **Assay ID** | ***Gene Symbol*** | **Gene Name(s)** |
| --- | --- | --- |
| Hs00153936_m1 | *ACAN* | aggrecan |
| Hs00953798_m1 | *ACVRL1* | activin A receptor type II-like 1 |
| Hs00192708_m1 | ADAMTS4 | ADAM metallopeptidase with thrombospondin type 1 motif, 4 |
| Hs01095524_m1 | ADAMTS5 | ADAM metallopeptidase with thrombospondin type 1 motif, 5 |
| Hs00181613_m1 | *ANGPT1* | angiopoietin 1 |
| Hs01550901_m1 | *ASPN* | asporin |
| Hs00234140_m1 | *CCL2* | chemokine (C-C motif) ligand 2 |
| Hs01011368_m1 | CCL20 | chemokine (C-C motif) ligand 20 |
| Hs00356601_m1 | *CCR2* | chemokine (C-C motif) receptor 2 |
| *Hs01890706_s1 | CCR6 | chemokine (C-C motif) receptor 6 |
| Hs00166657_m1 | *COL10A1* | collagen, type X, alpha 1 |
| Hs01097680_m1 | *COL11A1* | collagen, type XI, alpha 1 |
| Hs01028971_m1 | *COL1A2* | collagen, type I, alpha 2 |
| Hs00170014_m1 | *CTGF* | connective tissue growth factor |
| Hs00171022_m1 | CXCL12 | chemokine (C-X-C motif) ligand 12 |
| *Hs01921207_s1 | CXCR1 | chemokine (C-X-C motif) receptor 1 |
| *Hs00607978_s1 | CXCR4 | chemokine (C-X-C motif) receptor 4 |
| Hs00155479_m1 | *CYR61* | cysteine-rich, angiogenic inducer, 61 |
| Hs00609791_m1 | *FABP4* | fatty acid binding protein 4, adipocyte |
| Hs00173503_m1 | *FRZB* | frizzled-related protein |
| Hs99999905_m1 | *GAPDH* | glyceraldehyde 3-phosphate dehydrogenase |
| *Hs00167060_m1 | *GDF5* | growth differentiation factor 5 |
| Hs00748445_s1 | *GJA1* | gap junction protein, alpha 1, 43kDa |
| *Hs99999909_m1 | *HPRT1* | hypoxanthine phosphoribosyltransferase 1 |
| Hs00174202_m1 | *IL7* | interleukin 7 |
| Hs00174103_m1 | IL8 | interleukin 8 |
| Hs00899658_m1 | *MMP1* | matrix metallopeptidase 1 (interstitial collagenase) |
| Hs01548728_m1 | *MMP2* | matrix metallopeptidase 2 (gelatinase A, type IV collagenase) |
| Hs00968308_m1 | *MMP3* | matrix metallopeptidase 3 (stromelysin 1, progelatinase) |
| Hs00942589_m1 | MMP13 | matrix metallopeptidase 13 (collagenase 3) |
| Hs00707120_s1 | *NES* | nestin |
| Hs00171458_m1 | NGF | nerve growth factor (beta polypeptide) |
| Hs00182120_m1 | *NGFR* | nerve growth factor receptor |
| Hs00176787_m1 | *NTRK1* | neurotrophic tyrosine kinase, receptor, type 1 |
| Hs00966522_m1 | *PDGFB* | platelet-derived growth factor beta polypeptide |
| Hs01115513_m1 | *PPARG* | peroxisome proliferator-activated receptor gamma |
| Hs00231692_m1 | *RUNX2* | runt-related transcription factor 2 |
| Hs00165814_m1 | *SOX9* | SRY (sex determining region Y)-box 9 |
| Hs00277762_m1 | *SPARC* | secreted protein, acidic, cysteine-rich (osteonectin) |
| Hs00998130_m1 | *TGFB1* | transforming growth factor, beta 1 |
| Hs00610319_m1 | *TGFBR1* | transforming growth factor, beta receptor 1 |
| Hs00559661_m1 | *TGFBR2* | transforming growth factor, beta receptor II (70/80kDa) |
| Hs00171558_m1 | TIMP1 | TIMP metallopeptidase inhibitor 1 |
| Hs01091319_m1 | TIMP2 | TIMP metallopeptidase inhibitor 2 |
| Hs00927214_m1 | TIMP3 | TIMP metallopeptidase inhibitor 3 |
| Hs00900360_m1 | *TNFRSF11B* | tumor necrosis factor receptor superfamily, member 11b |
| *Hs01092186_m1 | *TNFSF11* | tumor necrosis factor (ligand) superfamily, member 11 |
| Hs00900058_m1 | *VEGFA* | vascular endothelial growth factor A |

*Also validated by Taqman qPCR

**SUPPLEMENTARY FIGURE LEGENDS**

**Supplementary Figure 1** CD271 cell distribution in BML sections assessed by immunohistochemistry. Light microscopy photomicrographs. (A) BML section showing cartilage fissuring and thinning with prominent subchondral CD271^+^ staining. (B) High-magnification image of rectangular area in (A) showing CD271^+^ staining within subarticular end-plate immediately beneath chondral lesion. Magnification bars: 500 μm (A), 200 μm (B).

**Supplementary Figure 2** Surface expression of CXCR4, CXCR1 and RANKL proteins by flow cytometry. Cultures were grown from magnetically-selected CD271^+^ cells. Live MSCs were gated as DAPI-negative, CD45^-^CD73^+^CD90^+^ cells. Histograms for representative cultures are shown for the different markers. (A) BML (empty histograms) and non-BML (filled histograms) donor-matched cultures and (B) MSCs cultures from HC, OP, MSC femoral heads. BML = bone marrow lesion; HC = healthy control; MSC = mesenchymal stem cell; OA = osteoarthritic; OP = osteoporotic.
