# Supplementary figures and images for: Mesenchymal Stem Cell Alterations in Bone Marrow Lesions in Patients With Hip Osteoarthritis
Source: Arthritis Rheumatol. 2016 Jun 24;68(7):1648–59. doi: 10.1002/art.39622 (PMC4941540; doi:10.1002/art.39622)

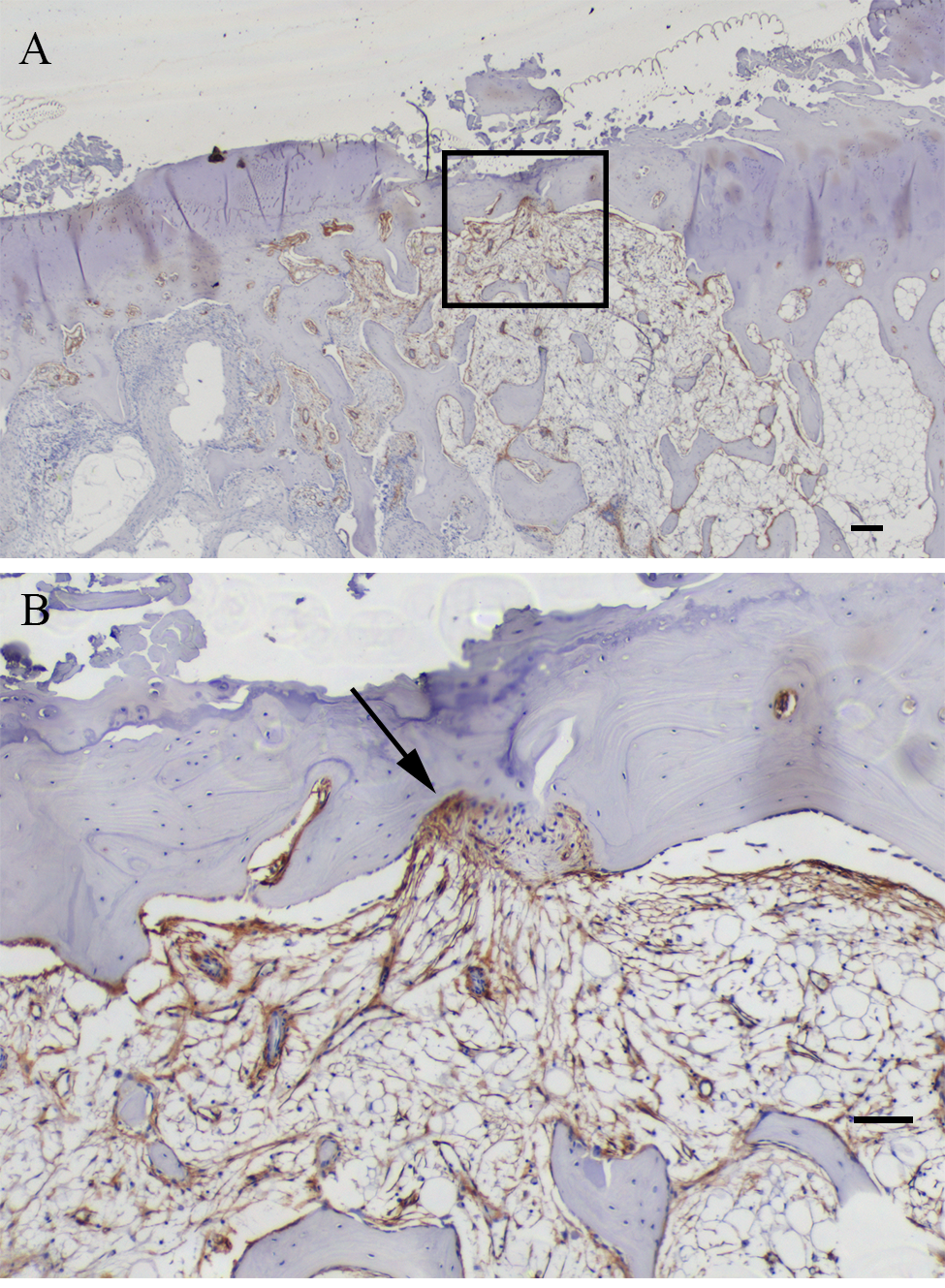

Supplement: Supplementary file 2 — Supplementary Figure 1 CD271 cell distribution in BML sections assessed by immunohistochemistry. Light microscopy photomicrographs. (A) BML section showing cartilage fissuring and thinning with prominent subchondral CD271+ staining. (B) High‐magnification image of rectangular area in (A) showing CD271+ staining within subarticular end‐plate immediately beneath chondral lesion. Magnification bars: 500 μm (A), 200 μm (B). [file ART-68-1648-s002.tif]

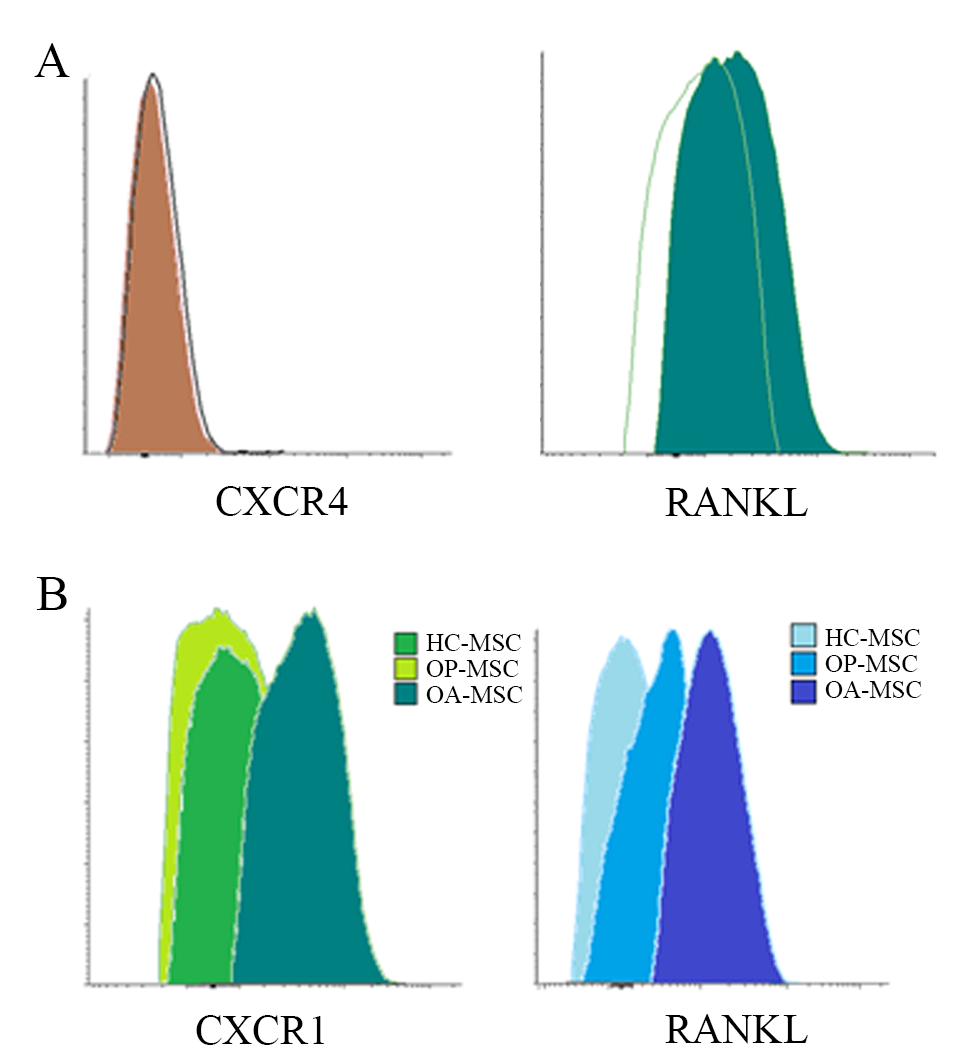

Supplement: Supplementary file 3 — Supplementary Figure 2 Surface expression of CXCR4, CXCR1 and RANKL proteins by flow cytometry. Cultures were grown from magnetically‐selected CD271+ cells. Live MSCs were gated as DAPI‐negative, CD45‐CD73+CD90+ cells. Histograms for representative cultures are shown for the different markers. (A) BML (empty histograms) and non‐BML (filled histograms) donor‐matched cultures and (B) MSCs cultures from HC, OP, MSC femoral heads. BML = bone marrow lesion; HC = healthy control; MSC = mesenchymal stem cell; OA = osteoarthritic; OP = osteoporotic. [file ART-68-1648-s003.tif]
